# Structure and thermodynamics of nonaqueous solvation by integral equation theory

**DOI:** 10.1186/1758-2946-4-S1-P6

**Published:** 2012-05-01

**Authors:** Roland Frach, Jochen Heil, Stefan M Kast

**Affiliations:** 1 Technische Universität Dortmund, Dortmund, 44227, Germany

## 

Electronic structure theory under the influence of apolar solvents suffers from substantial methodical difficulties since in this case the solvent-induced solute polarization originates mainly from specific directional interactions and higher electric multipoles. Continuum solvation models based on the dielectric solvent response such as the PCM approach ignore such interactions and can therefore not adequately model solvation effects in nonaqueous environments.

The “embedded cluster reference interaction site model” (EC-RISM) [1] retains the granularity of the solvent and represents a microscopically more detailed and therefore improved approach towards solvation modeling. EC-RISM is based on a self-consistent solution of solvent distribution functions described by a 3D integral equation theory and solute electronic structure by mapping the solvent charge distribution onto discrete, solute-embedding point charges. In aqueous solution EC-RISM theory is capable of calculating p*K*_a_ shifts [1] and tautomer ratios relatively fast and with high accuracy [2].

Here we outline the strength of the integral equation model by studying benzene and hexafluorobenzene solutions. In particular, the thermodynamics of differential solvation is quantified for organic compounds dissolved in these media. Moreover, it is shown that the respective solvent structures around particular solutes differ strongly, possibly leading to changes in the thermodynamic stability scale of various isomers which are not reproduced by the PCM model.
